# The *Uppsala APP* Mutation Promotes Wild‐Type Amyloid‐β Aggregation and Deposition In Vivo

**DOI:** 10.1002/advs.202514179

**Published:** 2026-03-31

**Authors:** Junyue Ge, María Pagnon de la Vega, Silvia Zampar, Enrica Cerilli, Srinivas Koutarapu, Ling Wu, Paul Fraser, Sertan Arkan, Vilmantas Giedraitis, Lars Lannfelt, Greta Hultqvist, Stina Syvänen, Martin Ingelsson, Jörg Hanrieder, Dag Sehlin

**Affiliations:** ^1^ Department of Psychiatry and Neurochemistry University of Gothenburg Gothenburg Sweden; ^2^ Department of Public Health and Caring Sciences Uppsala University Uppsala Sweden; ^3^ Krembil Brain Institute University Health Network Toronto Ontario Canada; ^4^ Tanz Centre for Research in Neurodegenerative Diseases University of Toronto Toronto Ontario Canada; ^5^ Department of Medical Biophysics University of Toronto Toronto Ontario Canada; ^6^ BioArctic AB Stockholm Sweden; ^7^ Department of Pharmacy Uppsala University Uppsala Sweden; ^8^ Departments of Medicine and Laboratory Medicine and Pathobiology University of Toronto Toronto Ontario Canada; ^9^ Department of Neurodegenerative Disease Queen Square Institute of Neurology University College London London UK; ^10^ Department of Neuropsychiatry Sahlgrenska University Hospital Gothenburg Sweden

**Keywords:** Alzheimer's disease, beta‐amyloid, mass spectrometry imaging, plaque pathology, Uppsala mutation

## Abstract

Amyloid‐β (Aβ) is widely regarded as a key initiator of theneurodegenerative cascade in Alzheimer's disease (AD).Studies of pathogenic mutations in the amyloid precursor protein (APP) genehave greatly advanced understanding of Aβ biochemistry, aggregation, anddeposition. One such mutation, Uppsala APP (APPUpp), produces AβUpp42_Δ19‐24_, whichis highly aggregation‐prone due to a six‐amino‐acid deletion in its central region.In both human APPUpp carriers and the recently developed tg‐UppSwe mouse model, Aβ depositspredominantly consist of the human AβUpp mutant.However, whereas human carriers produce both wild‐type Aβ (Aβwt) and AβUpp, tg‐UppSwe mice express only AβUpp. To better mimic the human condition, weinvestigated the pathological interplay between Aβwt and AβUpp using in vitroco‐aggregation assays and in vivo analyses in abitransgenic mouse model generated by crossing tg‐UppSwe with tg‐Swe mice. ELISA, immunohistochemistry, and MALDI mass spectrometry imaging revealed that earlydeposition of AβUpp42_Δ19‐24_accelerates aggregation and deposition of Aβwt species (Aβwt38, Aβwt40, Aβwt42), likely through a seeding or catalytic mechanism. Notably, bitransgenic mice developed pronounced plaque‐associated gliosisan alteration absent in tg‐UppSwe animals. These findings suggest a synergistic interaction betweenAβUpp and Aβwt that may influence onset, progression, and structural featuresof Aβ plaques in APPUpp mutation carriers.

## Graphical abstract

We investigated in vivo cross‐seeding of amyloid‐β (Aβ) isoforms in transgenic mice co‐expressing wild‐typeAβ and the Uppsala‐mutant Aβ variant (AβUpp), lacking six central residues. Weleveraged MALDI‐MS imaging and hyperspectral microscopy to follow spatio‐temporalAβ deposition. The Uppsalamutation resulted in more rapid pathology onset with early plaques showinghigher amounts of AβUpp, suggesting it seeds early plaque formation.

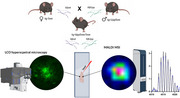

## 1 Introduction

Alzheimer's disease (AD) is characterized by deposition of amyloid‐β (Aβ) into parenchymal amyloid plaques and often also cerebral amyloid angiopathy (CAA) deposits in the brain vasculature. While Aβ is believed to initiate the disease process, downstream pathologies such as tau pathology and neuroinflammation are additional important drivers of neurodegeneration [1]. Disease‐causing mutations in the *APP* gene result in early‐onset forms of familial AD (FAD). Whereas many such mutations reside outside the Aβ region, affecting the production of Aβ, some pathogenic mutations are located inside the Aβ sequence. Most notably, the *Arctic APP* mutation generates a peptide that increases Aβ aggregation [2, 3, 4]. We recently identified and characterized yet another disease‐causing intra‐Aβ mutation, the *Uppsala APP* (*APPUpp*) mutation, which results in a six amino acid deletion in the middle of the Aβ sequence [5]. This mutation increases β‐secretase cleavage and thereby the production of AβUpp_Δ19‐24_. It also alters the anti‐amyloidogenic α‐secretase cleavage, which leads to the production of an N‐truncated peptide, AβUpp5‐42_Δ19‐24_, that was found in plaques from the *APPUpp* brain and therefore is assumed to contribute to the pathogenesis. Finally, and importantly, AβUpp42_Δ19‐24_ variants are prone to rapidly form fibrils that appear to have distinct structural features [5, 6].

Transgenic mouse models based on *APP* mutations have been crucial, not only to understand biomolecular mechanisms that are relevant to AD, but also to evaluate various treatment strategies. A large number of such models have been created (reviewed in [7]). Murine Aβ does not seem to contribute to plaque formation in these models, meaning that their Aβ pathology typically consists of human Aβ only [8, 9]. However, as the majority of pathogenic *APP* mutations described to date are causing disease in a heterozygous fashion, mutated forms of Aβ in patient brains are aggregating in an environment where wild‐type Aβ (Aβwt) is present. The co‐existence of the two Aβ variants may lead to a different aggregation behavior compared to if either form is present on its own. It is therefore important to study not only the disease‐causing mutant, but also the naturally occurring combination of Aβwt together with the Aβ mutant.

The tg‐Swe model, harboring the *Swedish* *APP* mutation *(APP KM670/671NL)*, is associated with a late onset of plaque pathology starting at about 10–12 months [4, 10], but displays abundant Aβ pathology at later stages. The *Swedish* *APP* mutation is localized just outside the N‐terminal end of Aβ and leads to increased production of human Aβwt. The tg‐Swe plaques are large, with a combination of dense and diffuse deposits, dominated by Aβ1‐38 and Aβ1‐40 but also with a significant contribution of Aβ1‐42. Similar to Aβ pathology in the AD brain, tg‐Swe plaques are surrounded by activated microglia and reactive astrocytes [10, 11]. The more recently developed tg‐UppSwe model harbors the *Uppsala APP* mutation (*690‐695Δ*) combined with the *Swedish APP* mutation, resulting in increased production of human AβUpp_Δ19‐24_. A previous study showed that plaques, which start to develop already at 4–6 months in this mouse model, are small, diffuse, and almost exclusively composed of AβUpp1‐42_Δ19‐24_, while lacking the typical Aβ‐associated gliosis [6]. In the human *APPUpp* brain, although AβUpp1‐42_Δ19‐24_ is the most abundant species, Aβwt peptides also seem to be present in the deposits. While microgliosis has not been assessed in this brain, reactive astrocytes are present in association with plaques [5]. These observations imply that the various Aβ peptides could affect each other in the aggregation process and further, that glial cells may respond differently to Aβ deposits of different structure.

To study the potential co‐deposition of Aβwt and AβUpp, and thereby simulating the situation in the brain of an *APPUpp* mutation carrier, we performed co‐aggregation studies both in vitro, using synthetic peptides mixed at different proportions, and in vivo, by crossing mice from the tg‐UppSwe and tg‐Swe lines [3, 4, 12].

## 2 Methods

### 2.1 Synthetic Aβ and ThT Assay

For in vitro aggregation experiments, peptides were generated on an Applied Biosystems ABI433A peptide synthesizer using FMOC‐solid phase techniques and purified by C18 reverse phase HPLC with trifluroacetic acid (TFA) in the mobile phase. The content of the isolated peptides determined by LC‐MS and total amino acid analysis. The lyophilized synthetic peptides Aβwt1‐40, Aβwt1‐42, and AβUpp1‐42_Δ19‐24_ were dissolved in 6 M GuHCl for 30 min, then subjected to gel filtration on a Superdex 75 10/300 GL column in degassed elution buffer (20 mm sodium phosphate buffer, 200 µm EDTA, 0.02% NaN3, pH 8.00) for collection of the monomer peak of each Aβ preparation. The fractions were collected on ice and the peptide concentrations were measured using the NanoOrange Protein Quantitation Kit (ThermoFisher Scientific) following the manufacturer's instructions. Monomers were then mixed with thioflavin T (ThT) (20 µm) to a starting Aβ concentration of 3.5 µm and added in triplicate into a 96‐well plate (black, clear bottom, half area; corning 3881). To monitor fibrillization at different time‐points, fluorescence was measured at 448 nm (excitation) and 485 nm (emission) every 5 min during 90 h with a FLUOstar Omegaplate reader (BMG Labtech).

To form fibrils for seeding studies, 5 µm of monomeric AβUpp1‐42_Δ19‐24_ peptides were incubated in the SEC elution buffer at 37°C for 24 h. The fibrils were further diluted and added to a 96‐well black plate mixed at different proportions (10% and 25%) with the different fresh monomer fractions with ThT (20 µm). Fibrillization for the individual peptides was monitored as explained above. Aggregation curves are presented as % of the maximum ThT signal, using the plateau phase of each curve as maximum. Each curve starts at minimum signal and ends when a maximum signal is reached. Each experiment was performed at least three times.

### 2.2 Mouse Models and Genotyping

**FIGURE 1:**
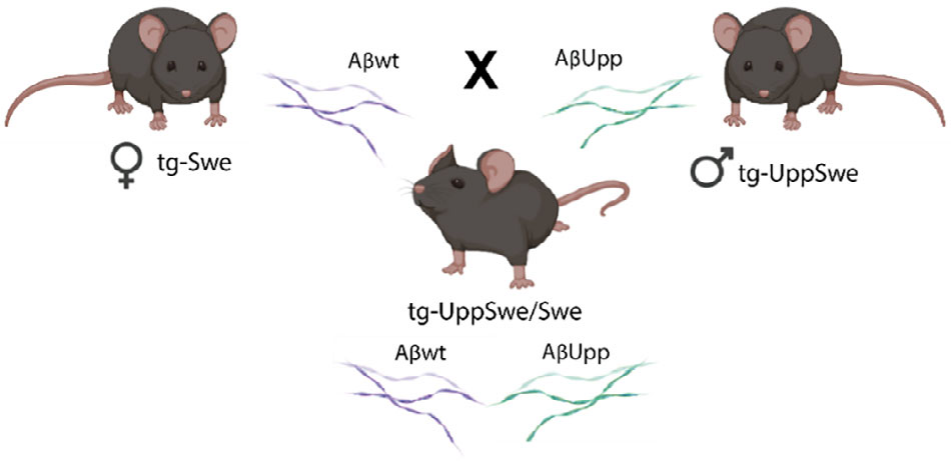
Breeding of tg‐Swe and tg‐UppSwe mice. Heterozygous tg‐Swe males were bred with heterozygous tg‐UppSwe females to produce tg‐UppSwe/Swe mice, heterozygous for each of the transgenes in the parent models. All mice were bred on a C57BL/6J‐BomTac background.

To study the in vivo co‐aggregation of Aβwt and AβUpp_Δ19‐24_, a cross was established between the two mouse models tg‐Swe [4] and tg‐UppSwe [6], heterozygously bred on a C57BL/6J‐BomTac background. Tg‐Swe females (*n* = 6) from the same breeding cage were mated with Tg‐UppSwe males (*n* = 3) from the same litter to produce the crossed mouse model tg‐UppSwe/Swe (Figure 1). During a period of four months, these nine breeding animals, housed into three cages, produced several litters with mixed genotypes and production of Aβ peptides: tg‐Swe (Aβwt), tg‐UppSwe (AβUpp), tg‐UppSwe/Swe (Aβwt and AβUpp) and wt, see Table 1 for APP expression and Aβ production properties of each model.

**TABLE 1:** APP expression and Aβ production in tg‐Swe, tg‐UppSwe, and tg‐UppSwe/Swe mice.

Mouse model	APP mutation	hAPP copy number	mAPP copy number	Aβ peptides
tg‐Swe	APP KM670/671NL	6	1	Aβwt (+ mAβ)
tg‐UppSwe	APP KM670/671NL, APP Δ690‐695	1	1	AβUpp (+ mAβ)
tg‐UppSwe/Swe	APP KM670/671NL, APP Δ690‐695	7 (6+1)	1	AβUpp+Aβwt (+ mAβ)

The mice were housed in an animal facility at Uppsala University, with free access to food and water, in rooms with controlled temperature and humidity under a 12 h/12 h light/dark cycle. At predefined ages, mice were euthanized and their brains were collected for further analysis of brain Aβ content and characteristics. The number, sex, and genotype of mice included in each age group are shown in Table 2. All experimental procedures were performed in accordance with the relevant guidelines and regulations. The study protocol was approved by the Uppsala Animal Experimental Ethical Committee (#5.8.18‐13350/2017). Mice were genotyped using two PCR reactions, both employing common forward primer (CATCCAAATGTCCCCTGCAT) and reverse primers specific for either hAPPwt, (TTGAACCCACATCTTCTGCAA) or hAPPUpp (ACCTTTGTTTGAACCCACCTT). Samples from the tg‐Swe mice were identified by the hAPPwt PCR, the tg‐UppSwe samples by the hAPPUpp PCR and the tg‐UppSwe/Swe samples by both assays. The DNA was extracted from ear tissue using KAPA DNA extraction kit. The PCR was performed using KAPA DNA polymerase and genotyping mix (ThermoFisher) in a final volume of 15 µL containing 30 ng genomic DNA and 0.2 µm of each primer. The following amplification protocol was used: denaturation for 5 min at 95°C followed by 30 cycles of 95°C for 45 s, 54°C for 45 s, and 72°C for 45 s and finished by final extension for 5 min at 72°C. Amplification products were analyzed on 1% agarose gel.

**TABLE 2:** Mice included in the present study.

	Tg‐Swe (female/males)	Tg‐UppSwe (female/males)	Tg‐UppSwe/Swe (female/males)	wt (female/males)
8 months	(0/2)	(1/1)	(1/1)	(1/1)
12 months	(1/3)	(2/2)	(2/2)	(2/2)
18 months	(2/2)	(3/1)	(5/1)	(3/1)

### 2.3 Tissue Preparation and Brain Homogenization

Female and male mice of all studied genotypes (Table 2) were sacrificed and perfused with 0.9% saline solution. Brains were isolated, dissected in half and immediately frozen on dry ice. The right hemisphere was used for cryosectioning. To study the molecular composition of Aβ pathology across the models, the cerebrum of the left hemisphere was homogenized using a Precellys Evolution (VRW, Stockholm, Sweden) (4 × 10s at 5500 rpm), and sequentially extracted in fractions of different solubility, ranging from the most soluble to the least soluble pool (TBS_100K_, TBS_16K_, TBST, SDS, FA). First, brain tissue was homogenized at a 1:5 weight/volume ratio in Tris‐buffered saline (TBS), then centrifuged at 16 000 × g for 1 h at +4°C to collect the supernatant (TBS_16K_). A fraction of the TBS16K extract was further centrifuged for 1 h at 100 000 × g to obtain TBS_100K_. The tissue pellet from TBS extraction was re‐homogenized according to the same procedure, first in TBS with 0.5% triton X (TBS‐T), then in 2% SDS, and finally in 70% formic acid (FA) to produce brain extracts containing Aβ of different solubilities.

### 2.4 Amyloid‐β ELISA

Total concentrations of Aβ1‐40 and Aβ1‐42 were analyzed by an ELISA method that does not distinguish between AβUpp_Δ19‐24_ and Aβwt. Ninety‐six‐well plates were coated with 1 µg/mL of anti‐Aβ40 (Agrisera, custom production) or anti‐Aβ42 (700254, Thermo Fisher) in PBS. For quantification of soluble Aβ aggregates, plates were coated with 1 µg/mL of the Aβ N‐terminal‐specific antibody 3D6 (in‐house produced). Plates were blocked with 1% BSA in PBS and standard series of synthetic Aβwt1‐40, Aβwt1‐42 or Aβ protofibrils (Innovagen, Lund, Sweden) were applied. Mouse brain extract fractions, diluted in ELISA incubation buffer (PBS, 0.1% BSA, 0.05% Tween‐20), were added to the plate in duplicates and incubated overnight at +4°C. Biotinylated 3D6 (binding to the N‐terminus of Aβ) (0.5 µg/mL) was added as secondary antibody for all ELISAs and signals were detected with streptavidin‐conjugated horseradish peroxidase (Mabtech AB, Nacka, Sweden) and K Blue Aqueous TMB substrate (Neogen Corp., Lexington, KY), before the plates were read with a spectrophotometer (Magellan infinite 200Pro) at 450 nm. Brain extracts from WT mice were used as negative controls.

### 2.5 TREM2 ELISA

TREM2 was quantified in TBS‐T brain extract from all mice included in the study (Table 2), using a previously described sandwich ELISA [13]. Briefly, 96‐well half‐area plates were coated overnight with 25 ng/well of anti‐TREM2 antibody AF1729 (R&D, Abingdon, UK), then blocked with 1% BSA in PBS for 2 h at RT. The brain extracts were diluted in ELISA incubation buffer and incubated overnight at 4°C, followed by detection with 0.25 µg/mL biotinylated anti‐TREM2 BAF1729 (R&D), HRP‐conjugated streptavidin and K Blue Aqueous TMB substrate and read at 450 nm with a spectrophotometer. A standard curve of recombinant TREM2 was used for quantification.

### 2.6 Immunohistostaining

For immunostaining, frozen brains were sagittally sectioned into 20 µm‐thick slices at −20°C, using a cryostat (Leica CM1860, Leica Biosystems, Nussloch, Germany). Sections were mounted on Superfrost Plus Adhesion Microscope slides. Sections were fixed overnight at 4°C in 4% paraformaldehyde (PFA), then rinsed with phosphate‐buffered saline (PBS).

For chromogenic staining, the sections were treated with pre‐heated citrate buffer, pH 6.3, for 30 min followed by 70% formic acid for 5 min. Aβ was visualized with anti‐Aβ40 (0.5 µg/mL; custom production, Agrisera, Umeå, Sweden), anti‐Aβ42 (0.5 µg/mL, Cat. No. 700254, Thermo Fisher Scientific, USA). For colorimetric staining the Vector NovaRED horse radish peroxidase (HRP) substrate kit (Vector Laboratories, Burlingame, CA) was used for detection.

For immunofluorescence, antigen retrieval was performed by incubating the sections for 30 min in antigen retrieval buffer (Tris/EDTA, 0.05% Tween‐20). After additional PBS washes, sections were incubated overnight at 4°C with rabbit anti‐Iba1 (1:500, Cat. No. 019‐19741, WAKO), mouse anti‐Aβ (1 µg/mL; 3D6 in‐house), and rabbit anti‐GFAP (1:500, Z0334, DAKO) primary antibodies diluted in blocking solution (1% bovine serum albumin [BSA], 0.3% Triton X‐100 in PBS). Following further PBS washes, sections were incubated for 1 h at room temperature with Alexa Fluor 488‐conjugated anti‐mouse IgG (1:500, Invitrogen) and Alexa Fluor 555‐conjugated anti‐rabbit IgG (1:500, Invitrogen) secondary antibodies. After final washes, sections were mounted using EverBrite Hardset Mounting Medium with DAPI (Biotium, Cat. No. 23004). Images were acquired using a Zeiss Observer Z.1 microscope equipped with a LD Plan‐Neofluar 40× objective (scale bar = 20 µm) and ZEN 3.7 software (Carl Zeiss Microimaging GmbH, Jena, Germany). All acquisitions were performed using constant parameters and were anonymized to avoid bias during both image acquisition and analysis.

Images were acquired in the full dynamic range (0–65535), stacked at identical intervals, and processed using Fiji. Background signal was estimated from unstained regions in each image and subtracted to ensure accurate comparison across samples. Thresholding was applied, and plaque regions of interest (ROIs) were delineated using the Analyze Particles function (size range: 5–∞). A minimum enclosing circle was generated for each plaque ROI using a Fiji macro. The macro extracted the ROI boundary coordinates and computed the smallest circle fully enclosing the plaque, defined by the midpoint and half distance of the two farthest boundary points. This circle was used as the standardized radial boundary for analysis. The density of IBA1^+^ and GFAP^+^ cells was quantified as the area of positive signal relative to the corresponding circle around each plaque.

### 2.7 Luminescent Conjugated Oligothiophenes (LCO) Imaging

Fluorescent amyloid microscopy was performed for 1) confocal microscopy and hyperspectral analysis as well as for 2) correlative MALDI/LCO imaging. Here, 12 µm thin cryosections were vacuum‐dried in a desiccator and fixed with ice‐cold absolute EtOH, 70% EtOH, and either 1) PBS or 2) Milli‐Q water for 10 min each. For hyperspectral LCO imaging, a double‐staining paradigm was used. Sections were incubated with tetrameric Formyl Thiophene Acetic Acid (2.4 µm q‐FTAA in PBS) and heptameric Formyl Thiophene Acetic Acid (0.77 µm h‐FTAA in PBS) [14, 15] for 30 min in the dark at RT. For correlative LCO/MALDI imaging, sections were incubated prior to MALDI matrix application with pentameric formyl Thiophene Acetic Acid (2.4 µm p‐FTAA in Milli‐Q water) for 30 min in the dark at RT [16]. Fluorescent microscopy images of p‐FTAA‐stained tissues were acquired using an Evo M3000 widefield microscope (Thermo Scientific).

For hyperspectral imaging, double LCO‐treated sections (q/h FTAA‐stained) were subsequently washed with PBS, mounted with a coverslip using Dako fluorescence mounting medium, and stored in dark conditions for a minimum of 24 h before light microscopy.

Hyperspectral imaging of double LCO‐stained transgenic mouse brain sections was performed using an inverted laser scanning confocal microscope (ELYRA PS.1 SIM/PALM LSM780, Zeiss, Germany), installed with a 32‐channel GaAsP spectral detector, in parallel spectral detection design, enabling simultaneous 32‐channel spectral readout in lambda mode. The confocal images were acquired with an excitation of 35‐nW, 458‐nm argon laser, with the Plan‐Apochromat 20×/0.8 objective. The continuous emission was acquired in the range of 405–700 nm. Regions on the tissue with plaque morphology were captured in lambda mode as a 32‐channel z‐stack image to elucidate the hyperspectral signatures.

Line scan analysis for hyperspectral differentiation on dual‐stained Aβ plaques was executed with the aid of an in‐house developed macro script in FIJI ImageJ. This macro allows the detection of wavelengths in a z‐stack image presenting the normalized intensity for each pixel in the region of interest across the plaques as a cross‐sectional emission profile.

### 2.8 MALDI‐MSI

For MALDI imaging, fresh frozen brains were cut into 12‐µm‐thick sections on a cryostat microtome (Leica CM 1520, Leica Biosystems, Nussloch, Germany) at −18°C, and thaw‐mounted on conductive ITO glasses (Bruker Daltonics, Bremen, Germany) [11]. Prior to MALDI analysis, frozen tissue sections were thawed and dried under vacuum for 15 min. A series of sequential washes of 100% EtOH (60 s), 70% EtOH (30 s), Carnoy's fluid (6:3:1 EtOH/CHCl_3_/acetic acid) (90 s), 100% EtOH (15 s), H_2_O with 0.2% TFA (60 s), and 100% EtOH (15 s) was carried out. For plaque visualization, tissues were stained with a fluorescent amyloid stain (LCO, p‐FTAA) followed by whole tissue microscopy imaging as described above using the Evos system.

Following microscopy, tissues were subjected to formic acid vapor for 20 min. 2,5‐Dihydroxyacetophenone (2,5‐DHAP) was used as matrix compound and applied using an HTX TM‐Sprayer (HTX Technologies LLC, Carrboro, NC, USA). A matrix solution of 15 mg/mL 2,5‐DHAP in 70% ACN/2% CH_3_COOH/2% TFA was sprayed onto the tissue sections using the following instrumental parameters: nitrogen flow (10 psi), spray temperature (75 °C), nozzle height (40 mm), eight passes with offsets and rotations, and spray velocity (1000 mm/min), and isocratic flow of 100 µL/min using 70% ACN as pushing solvent.

MALDI‐MSI experiments were performed on a rapifleX MALDI time‐of‐flight (TOF) Tissuetyper instrument (Bruker Daltonics). Measurements were performed at 10 µm spatial resolution, with the laser operating at a frequency of 10 kHz, a laser power of 90% (offset 3%, range 20%), and 200 shots per pixel. A mass range of 1500–6000 Da was analyzed in linear positive mode (LP). MALDI MSI acquisition was performed in flexImaging (version 5.1, Bruker Daltonics) [11].

### 2.9 Statistical Analysis

#### 2.9.1 MSI Data Image Segmentation

To identify the localization pattern associated with single plaques, each entire MALDI MSI dataset was interrogated through spatial segmentation using a bisecting k‐means‐based cluster analysis algorithm implemented in the SCiLS software (SCiLS Pro v 2023, Bremen, Germany) with the following parameters: Total Ion Count (TIC) normalization and medium smoothing strength. Segmented MSI cluster images were co‐registered with LCO‐stained images to identify plaque‐associated peptide patterns in the MSI data. Pseudo‐coloring was done by the software on a pixel‐to‐pixel basis and provided an initial visual representation of the chemical differences encoded in different pseudoclusters. Segmented plaque ROIs were exported as *.csv files for follow‐up univariate analysis. The plaque‐ROI mass spectral data were imported into Origin (v 8.1 OriginLab, Northampton, MA, USA) for baseline subtraction and peak picking using the implemented PeakAnalyzer function.

Statistical analyses of univariate data were performed using GraphPad Prism (version 6, San Diego, CA). Comparisons of three or more groups on a single dataset were performed by one‐way or two‐way ANOVA, followed by Tukey's post hoc test. A *p*‐value threshold of 0.05 was used for assessment of the statistical significance (non‐significant (*ns*), *p < 0.05, **p < 0.01, ***p < 0.001). Values are shown as means ± SD.

## 3 Results

### 3.1 AβUpp Peptides Seed Aβwt Aggregation In Vitro

**FIGURE 2:**
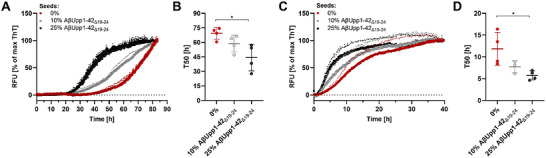
A) ThT assay displaying aggregation of Aβwt1‐40 alone or seeded with 10% or 25% of AβUpp1‐42_Δ19‐24_ fibrils. B) Time required to reach half the maximum ThT signal for the Aβ preparations in (A). C) ThT assay displaying aggregation of Aβwt1‐42 alone or seeded with 10% or 25% of AβUpp1‐42_Δ19‐24_ fibrils. D) Time required to reach half the maximum ThT signal (T50) for the Aβ preparations in (C). Data is presented as mean ± SD.

In the brain of *APPUpp* mutation carriers, both AβUpp and Aβwt are produced. While assuming that the in vivo proportion of Aβ40 and Aβ42 mimics their production ratio of approximately 10:1 [6], the faster aggregation of Aβ42 may give rise to elevated local concentrations of Aβ42 variants. To study if AβUpp_Δ19‐24_ can affect the aggregation of Aβwt in vitro, Aβ1‐40 wt and Aβ1‐42 wt monomers were therefore mixed with 10% or 25% pre‐formed fibrils of AβUpp1‐42_Δ19‐24_ and assessed with the ThT aggregation assay over time, in comparison with unseeded Aβ1‐40 wt and Aβ1‐42 wt. AβUpp1‐42_Δ19‐24_ significantly enhanced aggregation of Aβ1‐40 wt in a dose‐dependent manner (Figure 2A) and shortened the time required to reach half of the maximum ThT signal (Figure 2B). A similar, but less pronounced effect was seen for Aβ1‐42 wt (Figure 2C,D).

### 3.2 Aβ Plaque Pathology in Bitransgenic tg‐UppSwe/Swe Mice Differs from that of the Founder Lines

**FIGURE 3:**
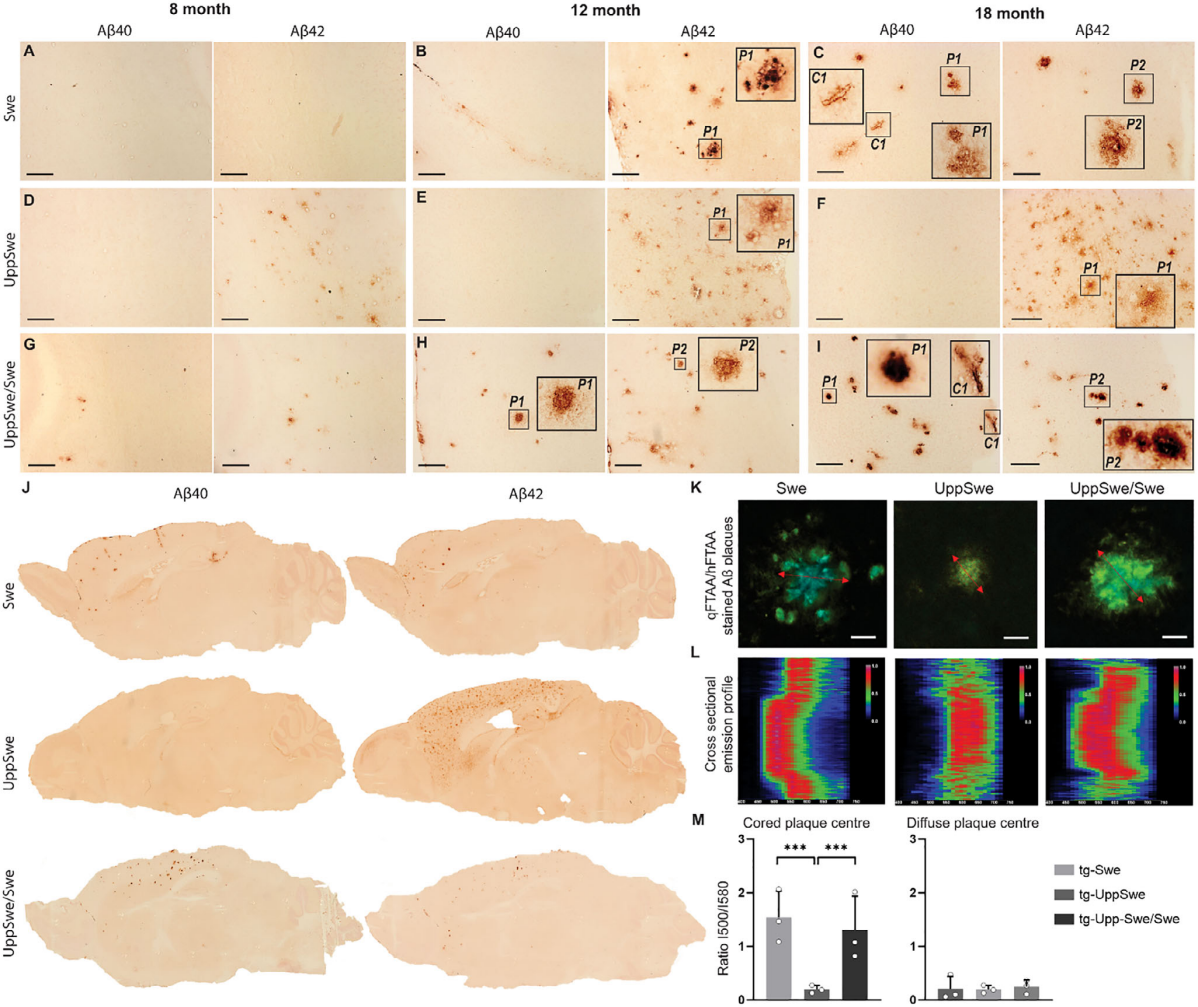
Representative Aβ40 and Aβ42 immunostaining of A–C) tg‐Swe, D–F) tg‐UppSwe and G–I) tg‐UppSwe/Swe mice at 8, 12, and 18 months of age. Insets show magnification of single plaques, identified by numbers (***P1‐P2***), and cerebral amyloid angiopathy, CAA (***C1***). Squares represent the regions displayed in the higher magnification images. Scale bars: 200 µm. J) Whole brain distribution of Aβ40 and Aβ42 in 18‐month‐old tg‐Swe, tg‐UppSwe, and tg‐UppSwe/Swe. K) Hyperspectral analysis on qFTAA/hFTAA dual‐stained Aβ plaques as captured from tg‐Swe, tg‐UppSwe, and tg‐UppSwe/Swe mice, respectively. Scale bar: 20 µm. L) Cross‐sectional emission profile of the plaques obtained by line scan analysis across Aβ plaques as indicated by the red double head arrows. M) LCO spectral statistics of the emission profiles from the different mouse lines comparing core vs diffused plaques. The data points in the graphs represent individual plaques (N = 2–4) sampled across *n* = 3 animals per mouse model. Data is presented as mean ± SD.

Brain tissue from the three mouse models tg‐Swe, tg‐UppSwe, and tg‐UppSwe/Swe was analyzed with Aβ immunostaining. Tg‐Swe mice lacked plaque pathology at the age of 8 months, while a few, mainly Aβ42 positive plaques appeared at the age of 12 months and 18‐month‐old mice displayed both Aβ40 and Aβ42 positive plaques (Figure 3A–C). The tg‐UppSwe brains showed Aβ42 positive plaques already at 8 months of age, with increased plaque density over time. However, no Aβ40 staining was observed at any age (Figure 3D–F). Unlike tg‐Swe mice, the crossed UppSwe/Swe line showed both Aβ40 and Aβ42 positive plaques already from the age of 8 months, which increased in size and apparent compactness as the mice aged (Figure 3G–I). Deposits present in the tg‐UppSwe brain appeared to be smaller and more abundant in number than those observed in the tg‐Swe and tg‐UppSwe/Swe brains. The plaques detected in the tg‐UppSwe/Swe line were larger and appeared to have more defined edges and contain larger amounts of Aβ per plaque compared to the other two mouse models (Figure 3C,F,I). In addition, the spatial distribution of plaque pathology was more widespread throughout the cortex in tg‐UppSwe mice, and at the oldest age it was also found in the hippocampus and thalamus. In tg‐UppSwe/Swe and tg‐Swe mice, the Aβ deposits were mainly concentrated in fewer cortical plaques, even at older age (Figure 3J). Vascular Aβ deposits, CAA, were primarily stained with the Aβ40 antibody and were abundant in both tg‐Swe and tg‐UppSwe/Swe at 18 months of age but could not be detected in tg‐UppSwe at any age (Figure 3C,F,I). LCO staining and spectral analysis confirmed that tg‐UppSwe plaques were predominantly of the diffuse type, while both tg‐Swe and tg‐UppSwe/Swe displayed both diffuse and cored plaques (Figure 3K–M).

### 3.3 Tg‐UppSwe/Swe Mice Develop Early Pathology Consisting of Both Aβ1‐40 and Aβ1‐42

**FIGURE 4:**
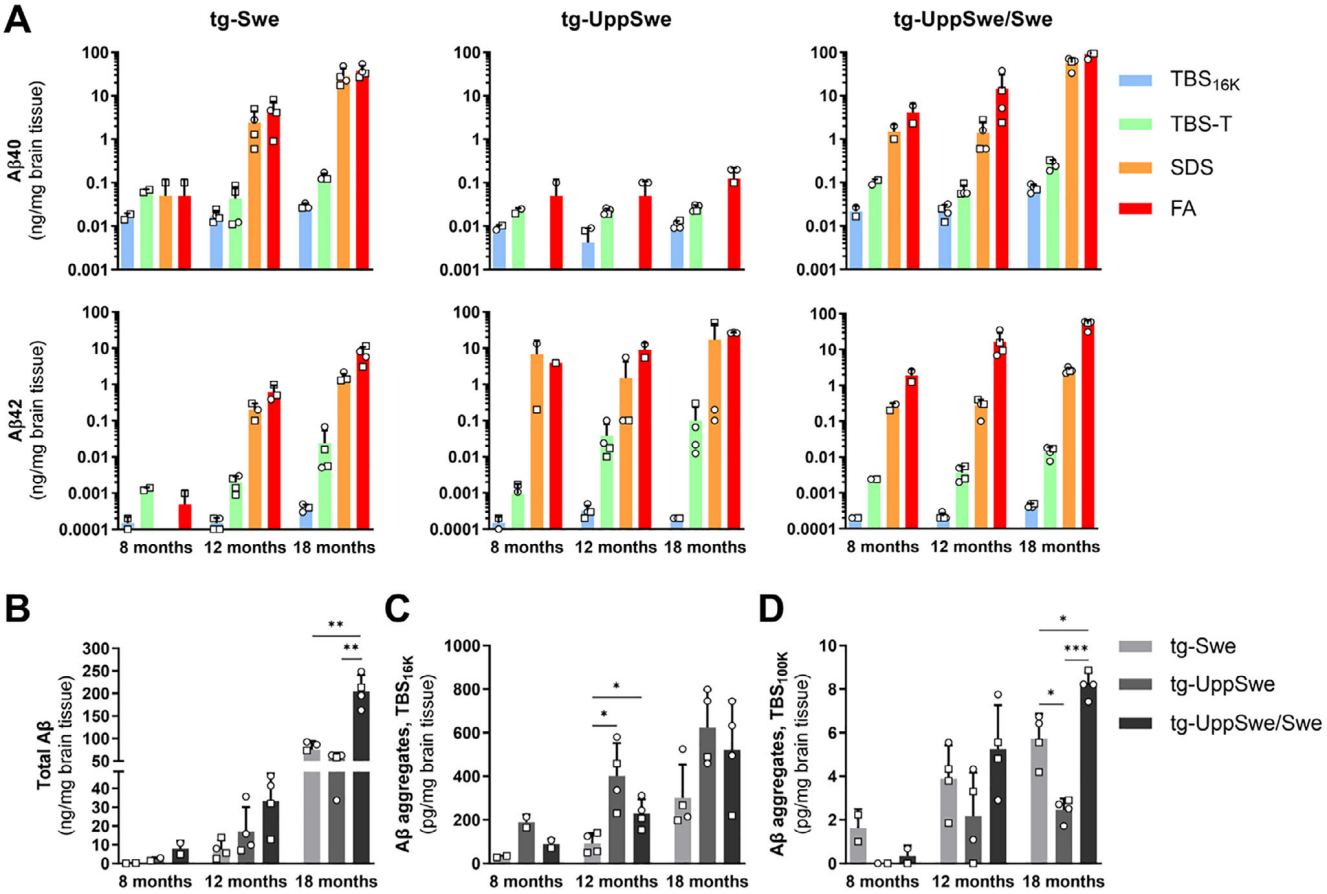
ELISA quantification of Aβ in brain extracts from tg‐Swe, tg‐UppSwe, and tg‐UppSwe/Swe mice at 8 (*n* = 2 per group), 12 (*n* = 4 per group), and 18 (*n* = 4 per group) months of age. A) Aβ1‐40 and Aβ1‐42 concentrations in brain tissue sequentially extracted in TBS_16K_ (blue), TBS‐T (green), SDS (orange), and FA (red). B) Total Aβ in brain tissue (sum of Aβ1‐40 and Aβ1‐42 in all fractions). C) Soluble Aβ aggregates in TBS_16K_ brain extract. D) Soluble Aβ aggregates in TBS_100K_ brain extract. Circles represent females and squares represent males. Data is presented as mean ± SD.

Total concentrations of Aβ1‐40 and Aβ1‐42 were analyzed in brain tissue extracts from the three mouse models, using an ELISA method that does not distinguish between AβUpp_Δ19‐24_ and Aβwt (Figure 4A). The TBS_16K_ fraction generally contained low levels of soluble Aβ1‐40 and Aβ1‐42 across all models, with little change over time, while the TBS‐T fraction of soluble and membrane‐bound Aβ displayed an age‐dependent increase in Aβ1‐42 with higher levels in older animals, especially in tg‐UppSwe mice.

Tg‐UppSwe mice displayed low levels of Aβ1‐40 in both the SDS and FA fractions, whereas Aβ1‐42 levels were elevated in both fractions already at 8 months (Figure 4A). In contrast, and in line with the histological examination, tg‐Swe mice displayed only background Aβ levels at 8 months, with increasing levels of both Aβ1‐40 and Aβ1‐42 from 12 to 18 months of age. As previously demonstrated, the tg‐Swe pathology was dominated by Aβ1‐40. Tg‐UppSwe/Swe mice showed an early build‐up of Aβ1‐42 in both the SDS and FA fractions already from 8 months of age, similar to what was observed in the tg‐UppSwe founder line. However, unlike both tg‐Swe and tg‐UppSwe, the tg‐UppSwe/Swe model also displayed elevated levels of Aβ1‐40 at 8 months, suggesting that the expression and deposition of AβUpp1‐42_Δ19‐24_ may initiate the aggregation and deposition also of Aβwt1‐40. Tg‐UppSwe/Swe mice also tended to display a higher FA/SDS ratio of both Aβ1‐40 and Aβ1‐42, suggesting a more compact plaque pathology (Figure 4A). Interestingly, 18‐month‐old tg‐UppSwe/Swe mice displayed significantly higher levels of total Aβ compared to both tg‐Swe and tg‐UppSwe mice. This amounted to almost 60% more than the sum of the two parent lines, suggesting that the combination of AβUpp and Aβwt accelerated Aβ pathology (Figure 4B). In addition, analyses of soluble Aβ aggregates were performed in TBS_16K_ and TBS_100K_ extracts, representing different levels of solubility. Levels of TBS_16K_ soluble aggregates were highest in tg‐UppSwe and lowest in tg‐Swe mice at all ages (Figure 4C), likely representing material from diffuse deposits in the brain. Levels of TBS_100K_ soluble aggregates on the other hand, were low in tg‐UppSwe mice, with significantly higher levels in both tg‐Swe and tg‐UppSwe/Swe at 18 months of age (Figure 4D). This is in line with the rapid fibrillation of the AβUpp1‐42_Δ19‐24_ peptide.

### 3.4 MALDI‐MSI Reveals Distinct Aβ Peptide Evolution Patterns in tg‐Swe and tg‐UppSwe Lines

**FIGURE 5:**
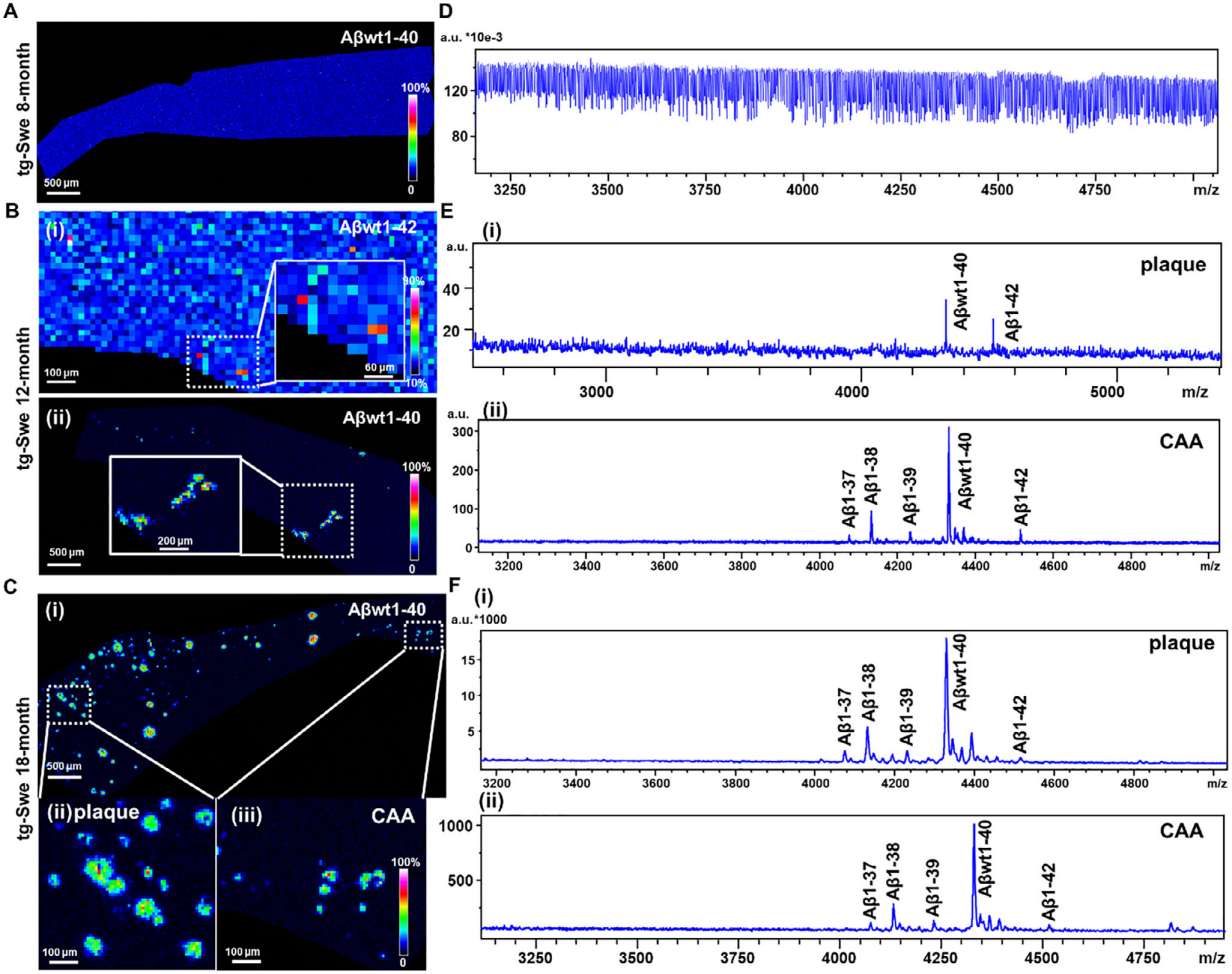
MALDI imaging of evolving peptide deposition in tg‐Swe mice. MALDI‐MSI single ion images (A–C) and plaque ROI spectra (D–F) of 8‐, 12‐, and 18‐month‐old tg‐Swe mice. A,D) No Aβ peptide was detected in 8‐month‐old mice. B,E) Single ion images (B) and spectra (E) of plaque (Ei) and CAA (Eii) in 12‐month‐old mice show differential content of 1–42. C,F) Single ion images (C) and representative spectra (F) of plaque (Cii and Fi) and CAA (Ciii and Fii) in 18‐month‐old mice.

To enable deeper studies of the composition and evolution of Aβ pathology in the different models, matrix‐assisted laser desorption/ionization mass spectrometry imaging (MALDI‐MSI) analyses were performed. Guided by LCO staining to identify Aβ plaques and CAA, this method allows to identify the exact peptide composition and also to distinguish between Aβwt, originating from tg‐Swe mice, and AβUpp_Δ19‐24_, originating from tg‐UppSwe. The pathology of tg‐Swe mice was too scarce to be analyzed at 8 months (Figure 5A,D), while 12‐month‐old mice showed both CAA and plaques. The plaques were primarily composed of Aβwt1‐40 and Aβwt1‐42 (Figure 5B‐i,E‐i), while CAA consisted primarily of Aβwt1‐40 and Aβwt1‐38, along with lower amounts of Aβwt1‐39 and Aβwt1‐37. In contrast to the plaque pattern, only relatively small amounts of Aβwt1‐42 were detected in CAA (Figure 5B‐ii,E‐ii). 18‐month‐old mice showed abundant parenchymal plaques composed mainly of Aβwt1‐40 and Aβwt1‐38, with a significant presence also of Aβwt1‐39 and Aβwt1‐37, as well as small amounts of Aβwt1‐42 (Figure 5C,F). The changes of peptide composition in plaques from 12‐ to 18‐month‐old mice indicates an age‐dependent diversification of Aβ species, suggesting a dynamic maturation process of Aβ pathology over time. Aβ1‐40 remained the most abundant species throughout, suggesting a central role in plaque deposition and structural maintenance in tg‐Swe.

**FIGURE 6:**
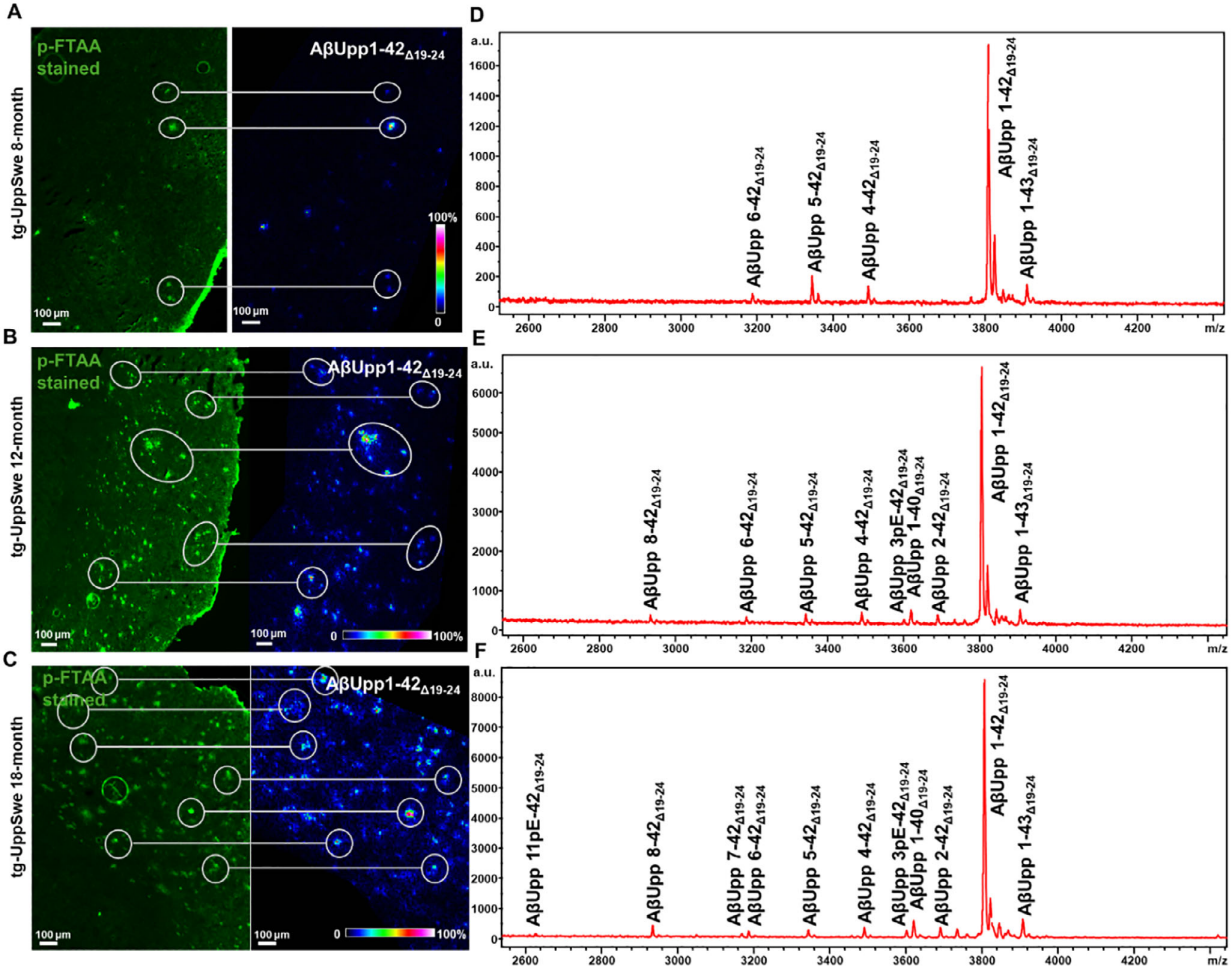
MALDI imaging of evolving peptide deposition in tg‐UppSwe mice. A–C) LCO (p‐FTAA) fluorescent images and correlative MALDI‐MSI single ion images. D–F) MALDI plaque ROI MS spectra of 8‐, 12‐, and 18‐month‐old tg‐UppSwe mice.

As expected from immunostaining and ELISA analyses, tg‐UppSwe brains contained small plaques which increased in abundance with age. At 8 months of age, tg‐UppSwe Aβ deposits mainly consisted of AβUpp1‐42_Δ19‐24_ peptides, with a significant contribution of the N‐terminally truncated peptides AβUpp6‐42_Δ19‐24_, AβUpp5‐42_Δ19‐24_, and AβUpp4‐42_Δ19‐24_, which may originate from a shifted α‐secretase cleavage of APPUpp. No AβUpp1‐40_Δ19‐24_ or AβUpp1‐38_Δ19‐24_ peptides were detected at this age (Figure 6A,D). By 12 months, AβUpp1‐42_Δ19‐24_ remained the predominant peptide, while additional truncated peptides, such as AβUpp3pE‐42_Δ19‐24_, and AβUpp1‐40_Δ19‐24_ began to appear in plaques (Figure 6B,E). In 18‐month‐old mice, both the number of plaques and the presence of truncated Aβ peptides continued to increase. Notably, AβUpp11pE‐42_Δ19‐24_ also began to appear in plaques (Figure 6C,F).

### 3.5 MALDI‐MSI Reveals Distinct Aβ Peptide Evolution Patterns in Bitransgenic tg‐UppSwe/Swe Compared to Monotransgenic tg‐Swe and tg‐UppSwe Lines

**FIGURE 7:**
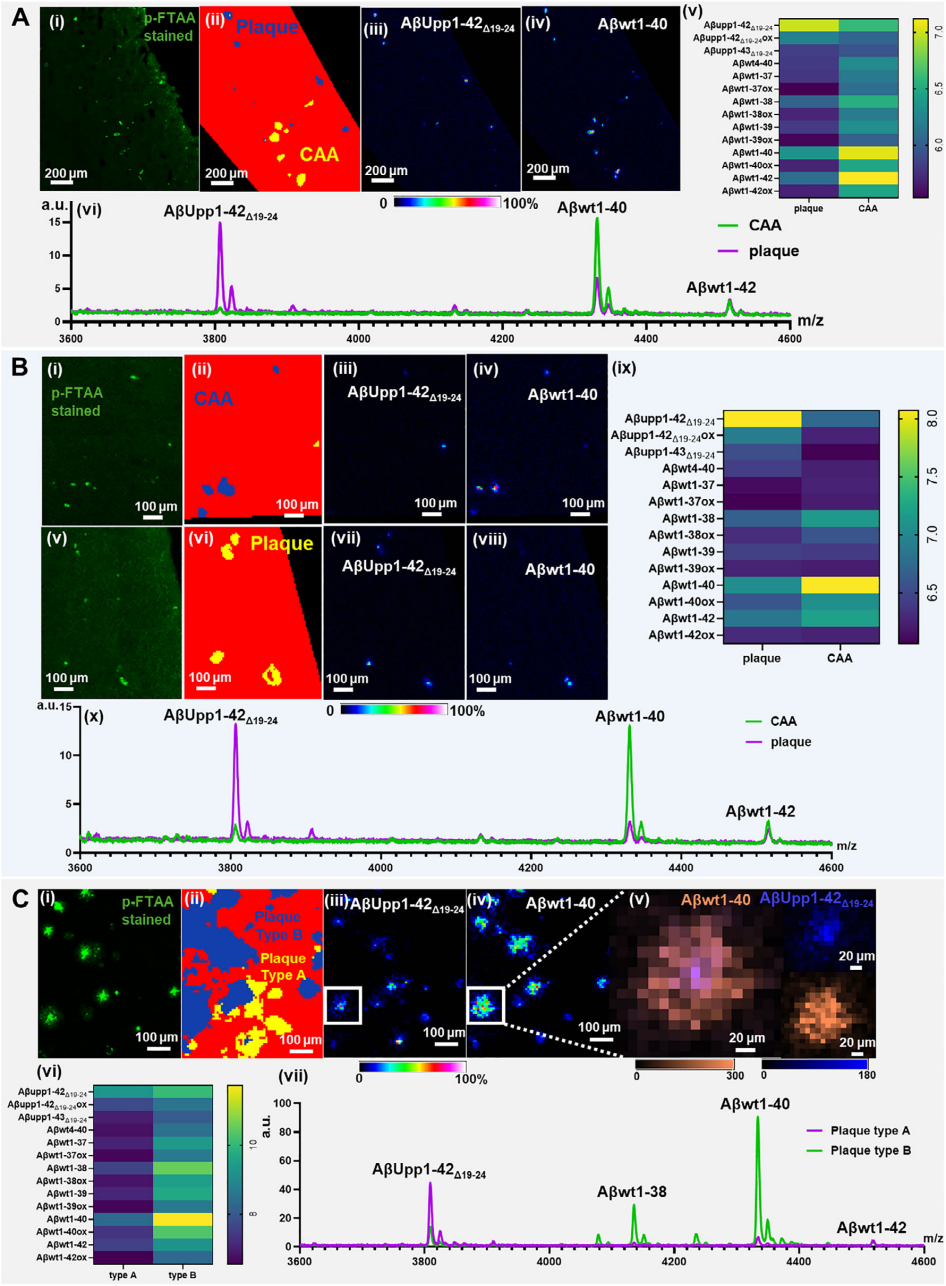
MALDI imaging of evolving peptide deposition in bitransgenic tg‐UppSwe/Swe mice A. LCO (A‐i) and hierarchical clustering analysis (HCA)‐based segmentation map (A‐ii) identified two clusters as plaque and CAA in 8‐month‐old tg‐UppSwe/Swe mice. Single ion images (A‐iii, iv), heat‐map of Aβ peptides (A‐v). and representative spectra of plaque and CAA (Avi) revealed distinct Aβ compositions in each at 8 months of age. B) LCO image (B‐i) and HCA‐based segmentation map (B‐ii) identified two clusters as plaque and CAA in 12‐month‐old tg‐UppSwe/Swe mice. Single ion images (B‐iii, iv) heat‐map of Aβ peptides (B‐v) and representative spectra of plaque and CAA (B‐vi) revealed distinct Aβ compositions in each at 12 months of age. C) LCO image (Ci) and HCA‐based segmentation map (C‐ii) identified two clusters of plaques in 18‐month‐old mice. Single ion images (C‐iii‐v), heat‐map of Aβ peptides (C‐vi) and representative spectra (C‐vii) of two subtypes of plaque revealed distinct Aβ compositions in each at 18 months of age. In the heat‐map, the Aβ peptides are arranged as follows: 1: AβUpp1‐42_Δ19‐24_; 2: AβUpp1‐42ox_Δ19‐24_; 3: AβUpp1‐43_Δ19‐24_; 4: Aβwt4‐40; 5: Aβwt1‐37; 6: Aβwt1‐37ox; 7: Aβwt1‐38; 8: Aβwt1‐38ox; 9: Aβwt1‐39; 10: Aβwt1‐39ox; 11: Aβwt1‐40; 12: Aβwt1‐40ox; 13: Aβwt1‐42; 14: Aβwt1‐42ox.

Following the time course MALDI analysis in the monotransgenic models, we then investigated how differential peptide deposition is affected in bitransgenic animals to evaluate the potential seeding effect of AβUpp on Aβwt species. Here, we observed that in tg‐UppSwe/Swe mice, Aβ plaques and CAA first appeared at 8 months of age. MALDI‐MSI data identified these structures as two distinct clusters using hierarchical clustering analysis (HCA) (Figure 7A‐i,ii). By analyzing Aβ peptide composition, we revealed that in plaques, AβUpp1‐42_Δ19‐24_ was the predominant peptide, with minimal presence of Aβwt peptides. In contrast, CAA was primarily composed of Aβwt1‐40 and Aβwt1‐42, with very low levels of AβUpp (Figure 7A‐iii,vi). Further HCA of CAA in 8‐month‐old mice identified two CAA subpopulations (Figure S1A). One subpopulation showed a high abundance of Aβwt1‐40, while the other was predominantly Aβwt1‐42 (Figure S1B,C). Notably, only a single CAA instance exhibited the second subpopulation with Aβwt1‐42 as dominance. In 12‐month‐old mice, MALDI‐MSI data still showed plaques and CAA as separate clusters (Figure 7B‐i and ii). Plaques remained dominated by AβUpp_Δ19‐24_, though Aβwt1‐40 accumulation was also observed. In CAA, Aβwt1‐40 was the predominant peptide, with low levels of AβUpp_Δ19‐24_ (Figure 7B‐iii,vi).

In brain tissues from 18‐month‐old tg‐UppSwe/Swe mice, large, round plaques were highly abundant. Here, segmentation analysis, using hierarchical clustering, identified two plaque clusters (Figure 7C‐i, ii). Plaque type A comprised smaller, apparently early‐stage plaques, in which AβUpp1‐42_Δ19‐24_ was the dominant Aβ species, with minimal presence of Aβwt1‐40 and Aβwt1‐42 (Figure 7C‐iii, vi). In contrast, plaque type B comprised larger, round plaques that primarily contained Aβwt1‐40, but with significant contribution from Aβwt1‐38 and smaller amounts of AβUpp1‐42_Δ19‐24_. These type B plaques appeared to be more mature and advanced in development showing dense Aβ fibrils compacted at the center of the plaque. This coexistence of distinct plaque morphologies and peptide compositions implies a possible seeding mechanism, where AβUpp1‐42_Δ19‐24_ may initiate plaque formation, while Aβwt1‐40 subsequently accumulates progressively to form the bulk of mature deposits. A comparison of representative data from the oldest age group of the three mouse lines is displayed in Figure S2.

### 3.6 Prominent Gliosis is Associated with Aβ Deposits in tg‐Swe and tg‐UppSwe/Swe, but not in tg‐UppSwe

**FIGURE 8:**
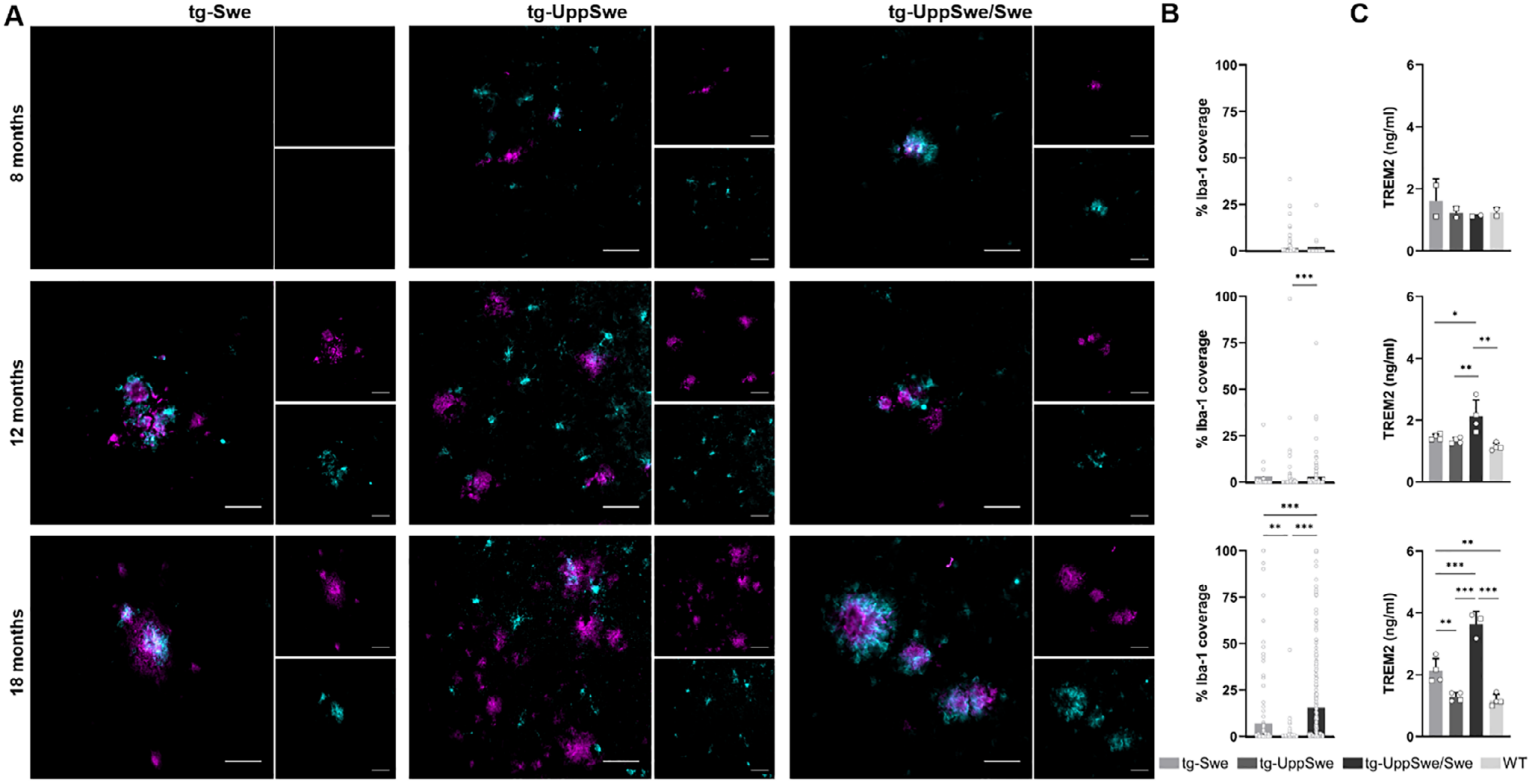
A) Aβ and microglial staining of cortical brain tissue from tg‐Swe, tg‐UppSwe, and tg‐UppSwe/Swe mice at 8, 12, and 18 months of age. The large images are merged single‐channel images of Aβ (3D6; magenta) and microglial (Iba‐1; cyan) staining, each shown separately on the side. Scale bar = 20 µm. B) Quantification of the percentage of Iba‐1 coverage around individual plaques from animals in (A). C) ELISA quantification of TREM2 levels in brain tissue extracts from groups of tg‐Swe, tg‐UppSwe, and tg‐UppSwe/Swe mice at 8 (*n* = 2 per group), 12 (*n* = 4 per group), and 18 (*n* = 4 per group) months of age. Circles represent females and squares represent males. Data is presented as mean ± SD.

Immunostaining of Aβ in combination with the microglial marker Iba‐1 showed microgliosis‐associated Aβ plaques in tg‐Swe mice at both 12 and 18 months of age, while no Aβ deposits were detected in younger tg‐Swe animals. In contrast, tg‐UppSwe showed abundant Aβ pathology already from 8 months of age, but with very limited co‐localization between Aβ and microglia at all ages. Tg‐UppSwe/Swe mice exhibited a few plaques at the ages of 8 and 12 months, where a subset of larger plaques were accompanied by microglia. At 18 months of age, extensive microgliosis was observed to surround the plaques in tg‐UppSwe/Swe brains (Figure 8A,B and Figure S3). Higher microglial coverage was found around large compared to small plaques (Figure S4). At the oldest age, microglia were also found around vascular deposits in both tg‐Swe and tg‐UppSwe/Swe mice (Figure S5). As a marker of overall microglial activation, the microglial protein TREM2 was quantified in brain extract from all three models at different ages. This analysis confirmed the pattern of microglial staining, with elevated TREM2 levels in 18‐month‐old tg‐Swe mice and increasing TREM2 levels in tg‐UppSwe/Swe mice from the age of 12 months. In tg‐UppSwe mice, TREM2 remained at the level of WT mice at all ages (Figure 8C)

**FIGURE 9:**
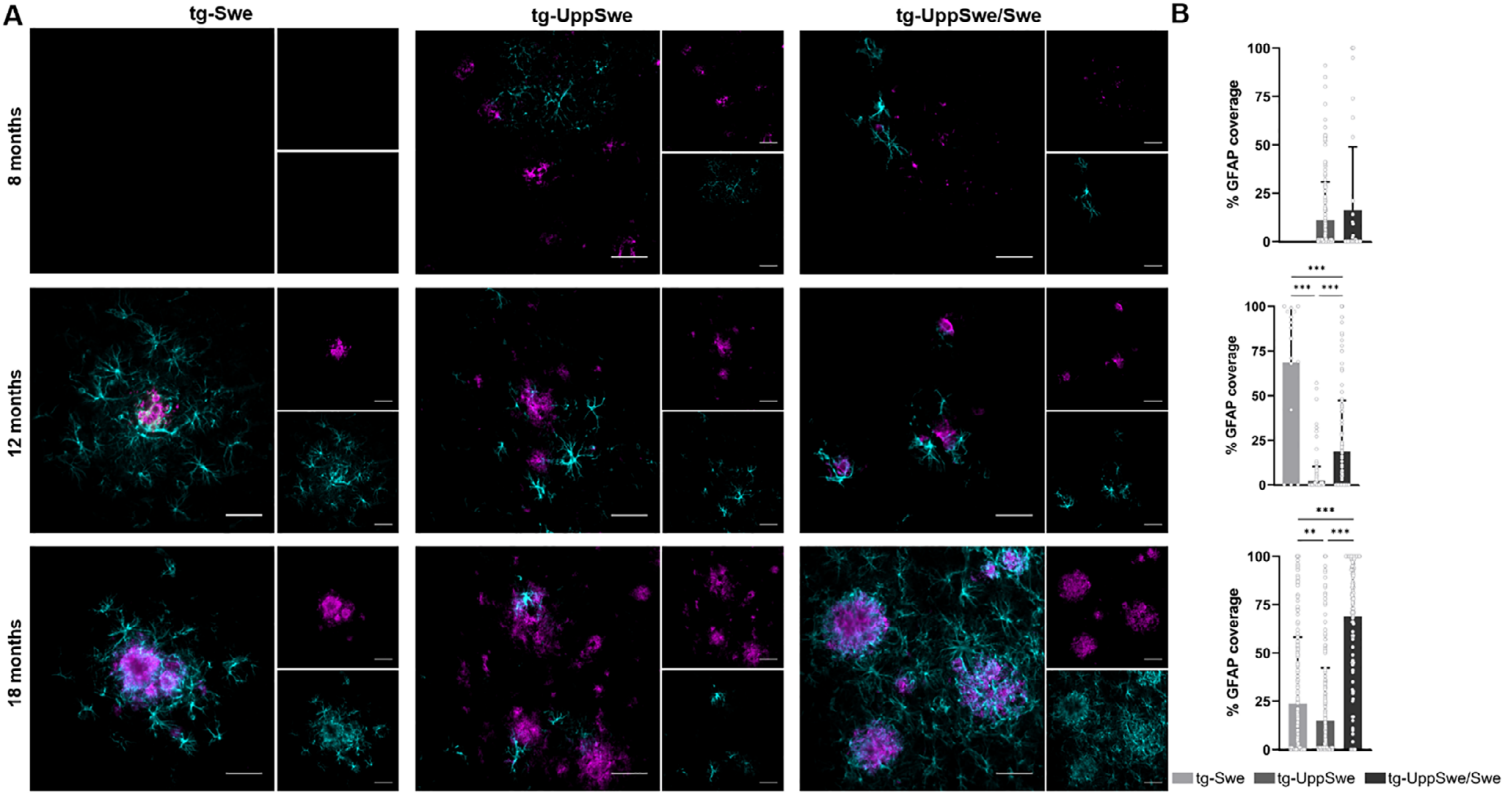
Aβ and astrocyte staining of cortical brain tissue from tg‐Swe, tg‐UppSwe, and tg‐UppSwe/Swe mice at 8, 12, and 18 months of age. The large images are merged single‐channel images of Aβ (3D6; magenta) and astrocyte (GFAP; cyan) stainings, each shown separately on the side. Scale bar = 20 µm. B) Quantification of the percentage of GFAP coverage around individual plaques from all animals in (A).

In addition, we investigated plaque‐associated astrocytes through glial fibrillary acidic protein (GFAP) immunostaining. Astrocytes displayed a similar plaque‐related pattern as microglia, with abundant clustering around the few plaques found in 12‐month‐old tg‐Swe mice and around the more abundant plaque pathology found in 18‐month‐old tg‐Swe mice. While astrocytes were present in tg‐UppSwe mouse brain, limited astrocyte clustering was found around the numerous small plaques in tg‐UppSwe mice at all ages. Similar to what was observed for microglia, tg‐UppSwe/Swe mice showed some astrocytes around the plaques at younger ages, with an increasing number of astrocytes surrounding plaques in old mice (Figure 9 and Figure S6). Both tg‐Swe and tg‐UppSwe mice had astrocytes surrounding vascular Aβ deposits (Figure S7).

## 4 Discussion

The *Uppsala APP* mutation generates AβUpp_Δ19‐24_ [5], a peptide that lacks part of the mid Aβ domain (KLVFFA) that has been identified as a driver of Aβ aggregation [17]. Still, AβUpp1‐42_Δ19‐24_ rapidly forms fibrils that adopt unique polymorphs in vitro and constitute the majority of Aβ in the parenchymal plaques found in *APPUpp* mutation carriers [5]. In line with this, plaques are formed mainly by AβUpp1‐42_Δ19‐24_ also in the recently developed tg‐UppSwe mouse model, which also recapitulates the altered β‐ and α‐secretase cleavages seen in *APPUpp* mutation carriers [6]. Contrary to the human condition, where the mutation is inherited in a heterozygous fashion, tg‐UppSwe mice produce human AβUpp_Δ19‐24_ in the absence of human Aβwt. As a consequence, several structural and histochemical differences can be found between the plaques formed in the human and transgenic mouse brain. For example, plaques in the human *Uppsala APP* brain are mainly ThS positive while plaques detected in the tg‐UppSwe mouse model are ThS negative [6]. Further, plaques found in the brain of human *APPUpp* mutation carriers are relatively large in size and appear dense, with a defined border, whereas tg‐UppSwe plaques are small and diffuse. Based on these observations, we hypothesized that the co‐existence of AβUpp_Δ19‐24_ and Aβwt, as in the human brain, could affect Aβ aggregation and the evolution of Aβ deposits. In support of this hypothesis, we found that the in vitro fibril formation of Aβwt was clearly affected by the addition of pre‐formed fibrils of AβUpp1‐42_Δ19‐24_. In a dose‐dependent manner, AβUpp1‐42_Δ19‐24_ fibrils accelerated the aggregation of Aβwt1‐40, shortening the lag phase and the time required to reach saturation. A similar, but less prominent, seeding was seen for Aβwt1‐42, which aligns with the faster inherent aggregation of this peptide. However, the results from these experiments could not clarify whether Aβwt and AβUpp peptides aggregated together or separately [18].

To model an in vivo environment similar to that in the human brain that harbors both AβUpp_Δ19‐24_ and Aβwt, tg‐UppSwe mice were crossed with tg‐Swe mice [4] to create the bi‐transgenic tg‐UppSwe/Swe model. Several differences in Aβ pathology emerged in the tg‐UppSwe/Swe mice compared to its founder lines. First, Aβ immunostaining of brains from tg‐UppSwe/Swe mice revealed that Aβ42 plaque pathology appeared already at 8 months, i.e., earlier than in the tg‐Swe mice. Interestingly, Aβ40 was also observed in the brain of these mice, and not exclusively Aβ42 as in tg‐UppSwe mice. This pattern was quantitatively supported by ELISA analyses of brain tissue extracts, demonstrating that Aβ1‐40 levels in 8‐month‐old tg‐UppSwe/Swe were equally high as in 12‐month‐old tg‐Swe. In addition, total Aβ levels in the oldest tg‐UppSwe/Swe mice were substantially higher than the combined levels of tg‐Swe and tg‐UppSwe mice. These observations suggest that the Aβ42 aggregates, supposedly carrying the *APPUpp* mutation accelerates the aggregation and deposition of Aβwt40 in the brain of tg‐UppSwe/Swe mice, either by acting as a seed for fibrillization or by catalyzing secondary nucleation [19]. Indeed, MALDI MSI analyses, which provide spectra of the exact peptide composition and abundance in a selected region of interest, confirmed that plaques appearing early, in 8‐month‐old tg‐UppSwe/Swe mice, were dominated by the AβUpp42_Δ19‐24_ peptide with small but significant contributions from Aβwt1‐40 and Aβwt1‐42. These young mice also displayed CAA, a feature that is absent in tg‐UppSwe mice at all ages and that appears at a later age in tg‐Swe mice. CAA in tg‐UppSwe/Swe mice was clearly dominated by Aβwt1‐40, with some contribution of Aβwt1‐42 and with barely detectable traces of AβUpp1‐42_Δ19‐24_. The deletion of residues 19–24 in the central hydrophobic domain in AβUpp1‐42_Δ19‐24_ may create an alternative seed nucleation center that facilitates the misfolding of Aβwt1‐40 peptides, promoting their deposition in parenchymal and vascular compartments. We thus speculate that despite the minimal content of the mutated peptide, it may have contributed to initiate early CAA deposition in young tg‐UppSwe/Swe mice, either by direct interaction or by affecting the cellular microenvironment in a way that promotes Aβ deposition. The Aβwt1‐40‐dominated CAA may also explain the relatively high levels of Aβ1‐40 measured with ELISA. In 12‐month‐old tg‐UppSwe/Swe mice, a similar pattern was observed, with increasing abundance of plaques that were still dominated by AβUpp1‐42_Δ19‐24_ and of CAA dominated by Aβwt1‐40. However, at 18 months of age, tg‐UppSwe/Swe mice presented two distinct types of plaques—type A, which were small and dominated by AβUpp1‐42_Δ19‐24_, and type B, which were large and round and mostly composed of Aβwt1‐40 and Aβwt1‐38, with significant contribution from AβUpp1‐42_Δ19‐24_ that appeared to be located in the center of some type B plaques. This again supports the hypothesis that diffuse aggregates of AβUpp42_Δ19‐24_ induce aggregation of Aβwt1‐40 and Aβwt1‐38 into separate homomolecular fibrils [18] that dominate the large, round, and defined, apparently mature plaques of type B that emerge at later disease stages in tg‐UppSwe/Swe mice. The small plaques that can still be seen in aged tg‐UppSwe/Swe mice, with AβUpp1‐42_Δ19‐24_ as the predominant peptide, may thus represent early‐stage plaques that are continuously formed throughout the aging process.

Several pyroglutamate‐modified Aβ peptides were found in the brain of the *APPUpp* mutation carrier [5], but among the mouse lines studied here, such modifications were only found in the brains of tg‐UppSwe mice. Pyroglutamation occurs in both vascular and parenchymal Aβ deposits and is known to enhance the ability of Aβ peptides to seed and aggregate, which thereby amplifies their neurotoxic effects [11, 20, 21]. However, in tg‐UppSwe mice, the plaques are diffuse and lack the compact fibrillar structure typically seen in cored plaques. This may suggest that pyroglutamation of AβUpp, in the absence of Aβwt, may not be sufficient to produce compact fibrillar plaques. Although the presence of pyroglutamate‐modified Aβ peptide species increases with age in this model, their exact role and impact on plaque development and toxicity remain unclear. Further investigation is therefore needed to clarify how pyroglutamated AβUpp peptides influence plaque characteristics and toxicity as the mice age. Interestingly, N‐terminally truncated variants of AβUpp42_Δ19‐24_ that are likely formed as a result of a shifted α‐cleavage in *APPUpp* mutation carriers [5], were here detected in Aβ plaques in the tg‐UppSwe mouse brain. Already from the age of 8 months, AβUpp5‐42_Δ19‐24_, accompanied by relatively lower amounts of both AβUpp4‐42_Δ19‐24_ and AβUpp6‐42_Δ19‐24_, were found in tg‐UppSwe Aβ deposits. This supports the notion that a shifted α‐cleavage contributes to AβUpp _Δ19‐24_ deposition. However, at older ages, the relative contribution of such truncated peptides decreased, suggesting that the majority of AβUpp_Δ19‐24_ is produced as full‐length peptides.

The plaques in 18‐month‐old tg‐UppSwe/Swe mice were equally compact as those seen in tg‐Swe mice, as confirmed by LCO stainings. The same analysis showed that tg‐UppSwe mice develop only diffuse plaques, but that are larger in number, as compared to tg‐UppSwe/Swe. At older age, tg‐UppSwe pathology also distributed over a greater area of the brain, including the hippocampus and thalamus. Thus, in addition to AβUpp42_Δ19‐24_ promoting Aβwt aggregation, the accumulation and deposition of AβUpp42_Δ19‐24_ seems to be affected by the presence of Aβwt. This results in fewer, but more condensed plaques with a defined border, similar to the plaques found in the brain of the human *APPUpp* mutaton carrier [5]. We speculate that this phenomenon may be mediated by glial cells, which have been reported to shape the morphology of Aβ plaques [22]. Previous studies demonstrated that tg‐UppSwe mice almost completely lacked plaque‐associated gliosis, despite a widespread Aβ deposition throughout the brain. In contrast, plaques in tg‐Swe plaques were consistently surrounded by both microglia and astrocytes. These findings were corroborated in the present study. Notably, in tg‐UppSwe/Swe mice, which develop plaques dominated by either AβUpp42_Δ19‐24_ or Aβwt40, glial involvement was restored. Interestingly, gliosis coincided with the formation of large Aβwt1‐40 positive plaques, sparsely occurring from 12 months of age and the markedly increasing with age. The abundant large plaques formed by predominantly Aβwt1‐38 and Aβwt1‐40 in aged tg‐UppSwe/Swe mice were associated with extensive gliosis. However, the subpopulation of smaller AβUpp42_Δ19‐24_ dominated plaques showed less glial involvement, suggesting that Aβwt is a necessary component to activate both microglia and astrocytes. Glial activation may thus occur upon maturation of type A into type B plaques in tg‐UppSwe/Swe mice, and potentially even play a role in the maturation process. We previously hypothesized that the low levels of soluble Aβ oligomers detected in aged tg‐UppSwe mice [6, 23] could represent the missing link required for microglial activation, potentially through interaction with TREM2 [6, 24]. Here, we found that soluble Aβ oligomer levels remained low across all ages in tg‐UppSwe mice, whereas both tg‐Swe and tg‐UppSwe/Swe mice exhibited increasing levels over time, along with increasing levels of TREM2. The dramatic increase in soluble Aβ oligomers occurring in 12‐month‐old tg‐UppSwe/Swe mice coincided with both Aβwt deposition and gliosis, supporting the hypothesis of oligomers activating a glial response. A recent report suggests that TREM2 binds to the first N‐terminal amino acid residues of Aβ aggregates [25], i.e., N‐terminally of the Uppsala deletion. However, although the structure of AβUpp42 fibrils obtained from tg‐UppSwe mice is very similar to human brain‐derived type I Aβ42 filaments [26], an important difference is that the flexible N‐terminal end of AβUpp fibrils starts at amino acid 7, leaving part of the TREM2 interaction site hidden in the rigid core of the Aβ42 fibril. We hypothesize that this structural feature of AβUpp42 aggregates may contribute the lack of interaction between AβUpp and microglia in a similar manner as it abolishes the binding of the anti‐Aβ antibody lecanemab (mAb158) to AβUpp aggregates in vivo [6, 26]. Another contributing factor to immune responses in the brain is the activation of T‐cells, which in the case of Aβ is mediated by interactions with amino acids 16–30 of the Aβ sequence [27]. This stretch of Aβ is largely missing in AβUpp, suggesting that Aβ processed by antigen presenting cells such as microglia and astrocytes may not activate T‐cells in the tg‐UppSwe mice.

A limitation of this study is the relatively small number of animals used, particularly in the two newly established AD mouse models. In addition, both male and female mice were included, which may introduce biological variability related to sex‐specific differences in Aβ deposition and disease progression. While this variability reflects real‐world heterogeneity and may enhance the generalizability of the findings, it could also reduce statistical power to detect subtle effects. Future studies with larger, sex‐balanced cohorts will be necessary to further validate and refine the observations presented here. Another potential limitation is associated with the introduction of transgenic human APP in mice. It has been reported that the endogenous murine APP may affect deposition of human Aβ [28]. However, as all transgenic models in this study share a similar design and harbor the same promoter, such effects should be comparable between the models.

In conclusion, the results of this study further illustrate that the pathogenetic effects of AβUpp can be recapitulated in transgenic mouse models and suggest that AβUpp/Aβwt interactions can explain the plaque pathology features in *APPUpp* mutation carriers. Mainly, the aggregation prone AβUpp1‐42_Δ19‐24_, with its rapid aggregation into uniquely structured fibrils, seems to be a potent driver of Aβwt aggregation and thereby co‐deposit into amyloid plaques that adopt a different structure. Mice expressing AβUpp/Aβwt pathology also develop abundant gliosis, which may be active in shaping the Aβ pathology.

## Funding

This work was supported by the Swedish Research Council (#2016‐02120, #2018‐02715, #2019‐02397, #2021‐03524, #2023‐02796); the National Institutes of Health (R01 AG078796, R21AG080705); Canadian Institutes for Health Research (PJT‐173479); Alzheimerfonden (AF‐1032753, AF‐980959); Hjärnfonden (FO2025‐0126), Konung Gustaf V:s och Drottning Victorias frimurarestiftelse, Stiftelsen Maja & J.P. Åhlén (#253045), Stiftelsen för Gamla Tjänarinnor (#2025‐329), Stohne's Stiftelse and Goljes stiftelse. This work was also supported by the Alzheimer Society of Canada and Krembil Foundation.

[Correction added on 2 April 2026 after first online publication: Funding information updated.]

## Conflicts of Interest

L.L. is a cofounder of BioArctic AB. M.I. is a paid consultant for BioArctic AB. The other authors declare no conflicts of interest.

## Supporting information




**Supporting File**: advs75038‐sup‐0001‐SuppMat.pdf.

## Data Availability

The data that support the findings of this study are available from the corresponding author upon reasonable request.
