# Supplementary material for: The Uppsala APP Mutation Promotes Wild‐Type Amyloid‐β Aggregation and Deposition In Vivo
Source: Adv Sci (Weinh). 2026 Mar 31;13(33):e14179. doi: 10.1002/advs.202514179 (PMC13271627; doi:10.1002/advs.202514179)
Supplement: Supplementary file 1 — Supporting File: advs75038‐sup‐0001‐SuppMat.pdf. [file ADVS-13-e14179-s001.pdf]

Supporting information

The *Uppsala APP* mutation promotes wild type amyloid-β aggregation and deposition *in vivo*

Junyue Ge[1*], María Pagnon de la Vega[2*], Silvia Zampar[4,5], Enrica Cerilli[2], Srinivas Koutarapu[1], Ling Wu[5], Paul Fraser[5,6], Sertan Arkan[2], Vilmantas Giedraitis[2], Lars Lannfelt[2,3], Greta Hultqvist[7], Stina Syvänen[2], Martin Ingelsson[2,4,5,8*], Jörg Hanrieder[1,9,10#*], Dag Sehlin[2#*]

[1] *Department of Psychiatry and Neurochemistry, University of Gothenburg, Gothenburg, Sweden*
[2] *Department of Public Health and Caring Sciences, Uppsala University, Uppsala, Sweden*
[3] *BioArctic AB, Stockholm, Sweden*
[4] *Krembil Brain Institute, University Health Network, Toronto, ON, Canada*
[5] *Tanz Centre for Research in Neurodegenerative Diseases, University of Toronto, Toronto, ON, Canada.*
[6] *Department of Medical Biophysics, University of Toronto, Toronto, ON, Canada*
[7] *Department of Pharmacy, Uppsala University, Uppsala, Sweden*
[8] *Departments of Medicine and Laboratory Medicine and Pathobiology, University of Toronto, Toronto, ON, Canada.*
[9] *Department of Neurodegenerative Disease, Queen Square Institute of Neurology, University College London, London, UK*
[10] *Department of Neuropsychiatry, Sahlgrenska University Hospital, Gothenburg, Sweden*

* Equal contribution
[#] Corresponding authors

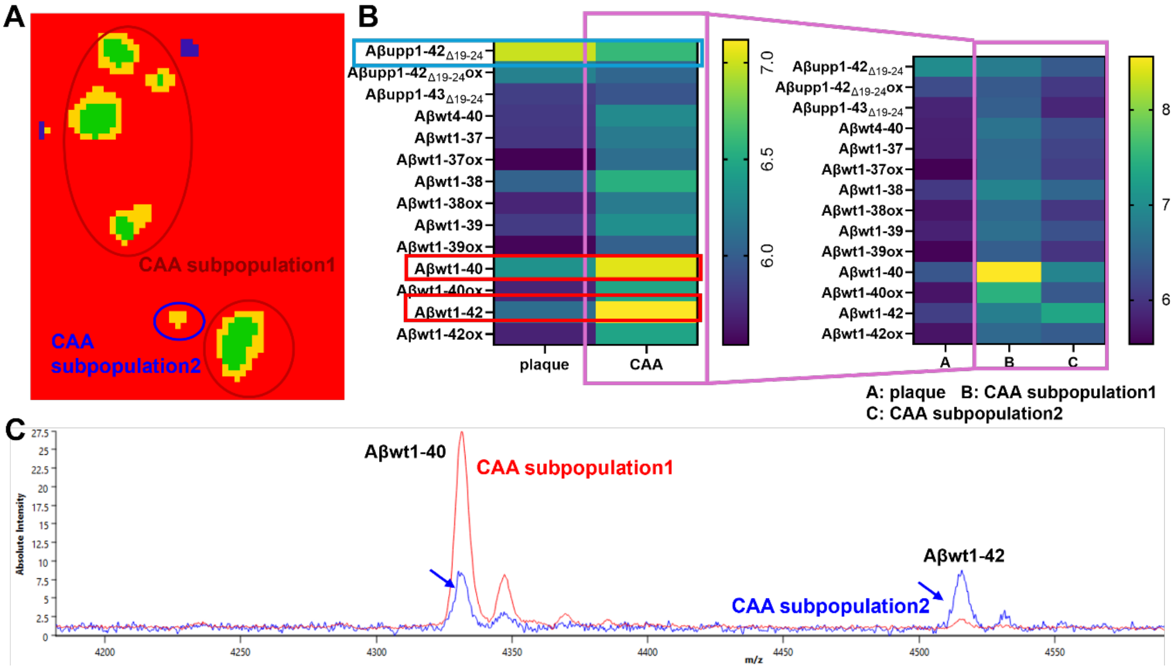

Figure S1. (**A**) HCA-based segmentation map revealed two subpopulations of CAA in 8-month-old tgUppSwe/Swe mice. (**B**) Heatmap of amyloid peptide compositions in two subpopulations of CAA. (**C**) Representative spectra of two subpopulations of CAA at 8-month-old.

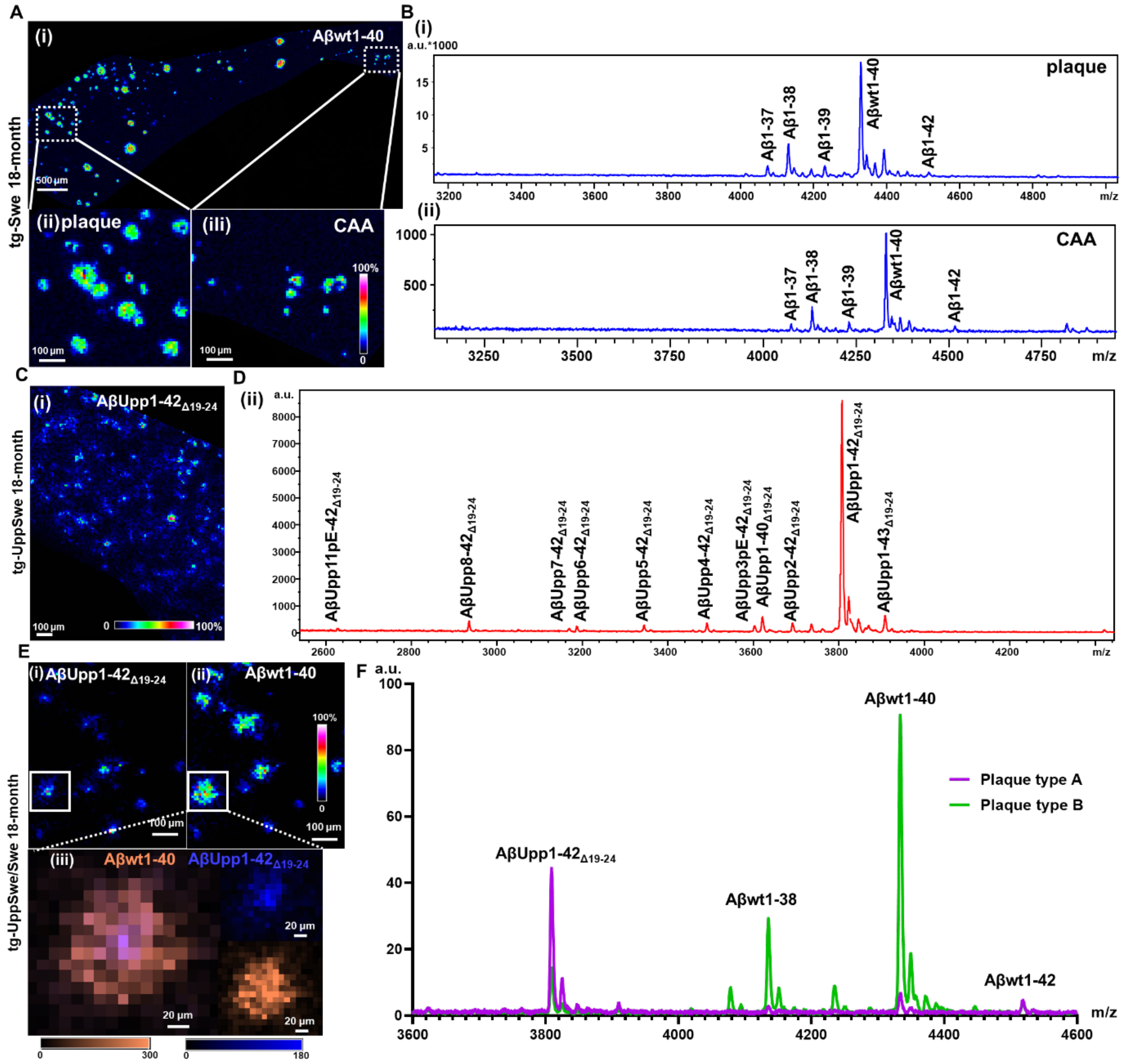

Figure S2. MALDI-MSI characterization of Aβ isoform heterogeneity and plaque pathology in transgenic AD mouse models at 18 months. Single ion images (A) and representative spectra (B) of plaque (Aii and Bi) and CAA (Aiii and Bii) in tgSwe 18-month-old mice. Single ion image (C) and representative spectra (D) of plaque in tgUppSwe 18-month-old mice. Single ion images (E) and representative spectra (F) of plaques in tgUppSwe/Swe 18-month-old mice.

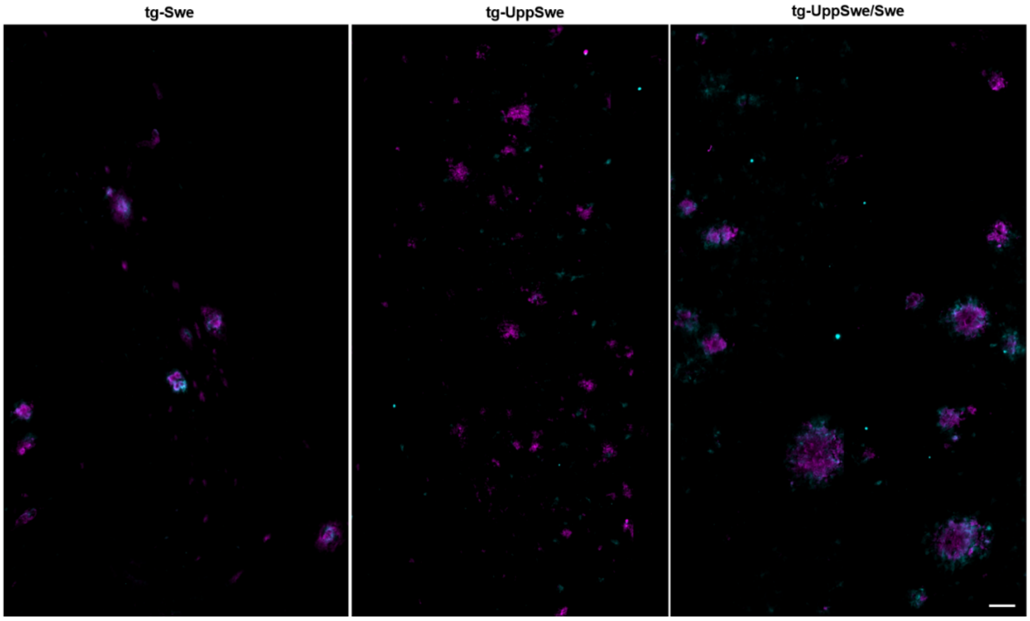

Figure S3. Aβ (3D6; magenta) and microglial (Iba-1; cyan) staining of cortical brain tissue from tg-Swe, tg-UppSwe and tg-UppSwe/Swe mice at 18 months of age at low magnification. Scale bar = 50 µm.

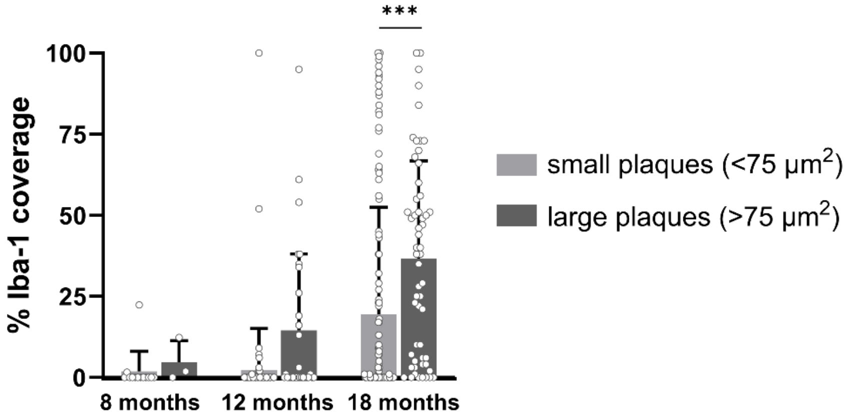

Figure S4. Quantification of the percentage of Iba-1 coverage around individual plaques from tg-UppSwe/Swe animals at 8, 12 and 18 months of age, divided in small (<75 µm²) and large (>75 µm²) plaques. Data is presented as mean ± SD.

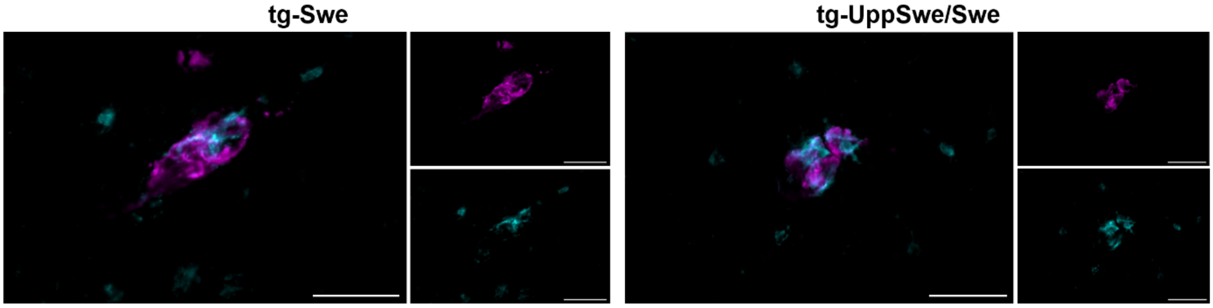

Figure S5. Aβ (3D6; magenta) and microglial (Iba-1; cyan) staining of vascular deposits in cortical brain tissue from tg-Swe and tg-UppSwe/Swe mice at 18 months of age. Scale bar = 50 µm.

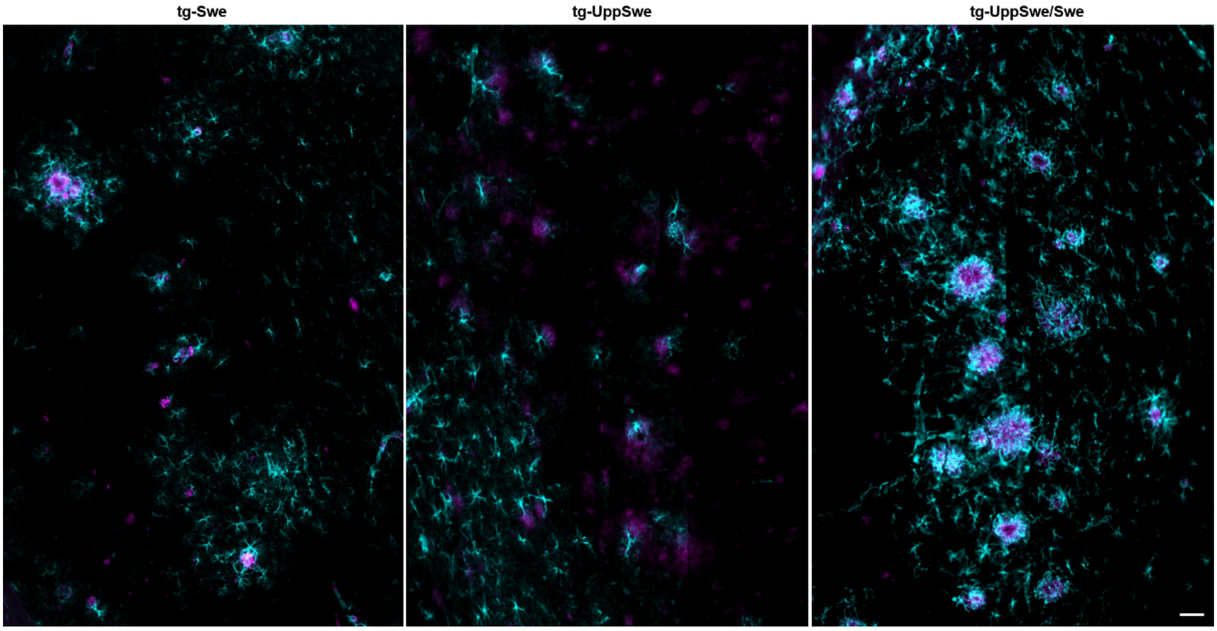

Figure S6. Aβ (3D6; magenta) and astrocyte (GFAP; cyan) staining of cortical brain tissue from tg-Swe, tg-UppSwe and tg-UppSwe/Swe mice at 18 months of age at low magnification. Scale bar = 50 µm.

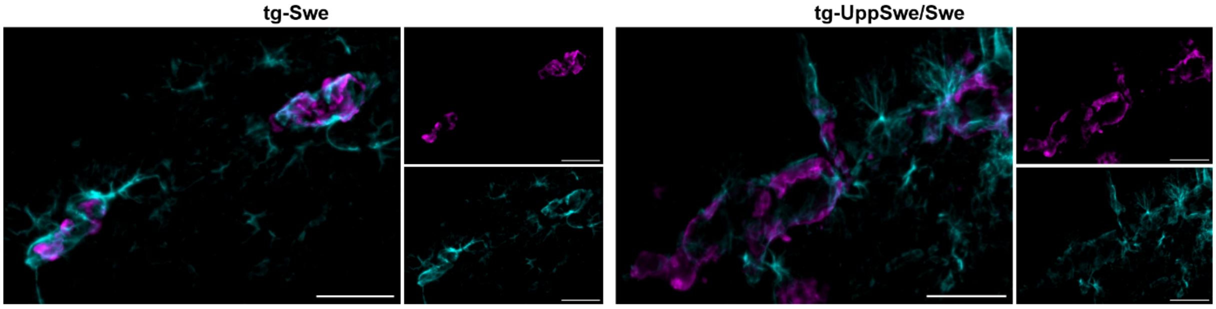

Figure S7. Aβ (3D6; magenta) and astrocyte (GFAP; cyan) staining of vascular deposits in cortical brain tissue from tg-Swe and tg-UppSwe/Swe mice at 18 months of age. Scale bar = 50 µm.
